# Evaluation of INI1 Protein Expression Through IHC Study in Pediatric High-Grade Brain Tumors in South of Iran in 2008-2021

**DOI:** 10.30699/IJP.2023.561858.297

**Published:** 2023-07-15

**Authors:** Maryam Mohebbi, Mansoureh Shokripour, Maral Mokhtari

**Affiliations:** Department of Pathology, Medical School, Shiraz University of Medical Sciences, Shiraz, Iran

**Keywords:** ATRT, CNS tumors, High grade, INI1, Pediatric

## Abstract

**Background & Objective:**

Brain tumors are the most frequent solid tumors in children. High-grade tumors are more challenging in diagnosis. Atypical teratoid rhabdoid tumor (ATRT) may be mistaken for other high-grade brain tumors. Molecular genetic analysis of ATRT has shown deletion and mutation in the *hSNF5/INI1* gene in most of the cases. The INI1 protein expression can be helpful for the accurate diagnosis.

**Methods:**

In this study, immunohistochemical staining (IHC) using INI1 antibody was performed to determine the possibility of ATRT misdiagnosis. Totally, 147 tumors including 6 ATRTs, 81 medulloblastomas, and 60 other CNS tumors were examined in children between 0 and 17 years old.

**Results:**

No nuclear staining was found in the six ATRT cases, while most of other CNS tumors demonstrated nuclear staining. Five cases were previously diagnosed with medulloblastoma, primitive neuroectodermal tumor (PNET), and anaplastic oligodendroglioma, while the diagnoses were changed to ATRT based on the re-evaluation of the H&E slides and INI1 study. Additionally, two cases were recurrent tumors whose features were consistent with those of ATRT. The INI1 immunostaining was negative in these cases.

**Conclusion:**

INI1 was very helpful in distinguishing ATRT from its mimickers in challenging cases. All known ATRT cases in this study were immunonegative for INI1. Thus, INI1 is recommended to be used in the initial IHC panel for the high-grade brain tumors, especially in children under the age of three years, so that they can benefit from intensified therapeutic regimens.

## Introduction

Brain tumors are the most frequent solid tumors and the second most common tumors in children. They are also the leading cause of cancer-related death in this population. Younger children are more involved with embryonal tumors such as medulloblastoma and atypical teratoid rhabdoid tumor (ATRT), while glial tumors are more prevalent in elder children (1, 2). Recently, novel information has become available regarding the genetics of the central nervous system (CNS) tumors. Moreover, newer antibodies that can be used in immunohistochemistry (IHC) are continuously being manufactured, some of which have diagnostic and/or prognostic applications (3, 4).

INI1 is a component of the SW1/SNF chromatin remodeling complex, which is encoded by the *INI1/hSNF5/SMARCB1/BAF47* locus at 22q11 and works in an ATPase-dependent fashion to remodel chromatin. INI1 is needed for both in vivo and in vitro chromatin remodeling activity of the SW1/SNF complex (3). ATRTs are molecularly identified by the biallelic inactivation of the *INI1/SMARCB1* gene, as evidenced by the loss of INI1 nuclear expression in IHC (5, 6). Rare ATRTs have the loss of *SMARCA4* (*BRG1*), as another SWI/SNF member (2, 7). Thus, not all ATRTs are immunonegative for INI1 and approximately 2% show retained INI1 (8). High-grade embryonal tumors can differentiate along many divergent lineages, as there is a spectrum of diseases including neuronal, astrocytic, melanocytic, and muscular disorders (9). The INI1-negative embryonal tumors have aggressive behavior and poor outcome if treated by the conventional therapy regimens. Therefore, detection of ATRTs is very important. In addition, these tumors may benefit from intensified treatment (10). 

Misclassification of these tumors as other embryonal tumors can happen due to their complex and varied histological patterns as well as the rarity of the tumor and polyphenotypic immunoprofiles (4, 11). According to the World Health Organization (WHO) 2016 and 2021 classification, diagnosis of ATRTs requires INI1 confirmation and tumors without INI1 loss should be reported as CNS embryonal tumors with rhabdoid features. Besides, PNET entity no longer exists (12, 13). 

The present study aims to evaluate the INI1 nuclear expression in different types of high-grade brain tumors to rule out any possibility of ATRT misdiagnosis. Considering the fact that the prognosis of ATRT is much worse than other high-grade brain tumors and needs more intensified chemoradiation therapy (14), and since divergent differentiations in this tumor can mislead pathologists (11), this study assessed the role of INI1 IHC in the diagnosis of ATRT in Shiraz Province patients, southern Iran.

## Material and Methods

**Re-evaluation of Previously Diagnosed High-Grade Brain Tumors**

In this study, 147 pediatric cases (0-17 years old) with the original pathological diagnosis of medulloblastoma (n=84), ATRT (n=6), glioblastoma multiform (GBM, n=29), anaplastic astrocytoma (n=4), anaplastic ependymoma (n=8), anaplastic ganglioglioma (n=1), medulloepithelioma (n=2), embryonal tumor, not otherwise specified (NOS) (n=4), pinealoblastoma (n=2), choroid plexus carcinoma (n=1), primitive neuroectodermal tumor (PNET, n=3), embryonal tumor with multilayered rosettes (n=1), mixed glial tumor (n=1), and undifferentiated high-grade tumor (n=1) were retrospectively reviewed from the pathology archives in Shiraz Nemazee Hospital from 2008 to 2021. This was done to rule out the presence of the rhabdoid cell component within the tumors that might have been missed during the routine diagnosis and review process.

**Re-classification of Tumors According to WHO’s 2021 Classification**:

A total of three cases were previously diagnosed as PNET (n=3), recalsiified to the CNS embryonal tumor, NOS (n=2) and ATRT (n=1) based on the WHO new classification.

**Immunohistochemistry**

The most typical formalin-fixed paraffin-embedded (FFPE) tissues of 4 µm Thickness were used for the immunohistochemical staining on 126 cases. These cases were manually stained by our primary INI1 [BAF47/SNF5], clone 25/BAF47 mouse monoclonal antibody (Master Diagnostica, Selliva, Spain). A commercially available biotin streptavidin immune peroxidase kit was employed for coloration. Unfortunately, other blocks could not be retrieved. 

**Statistical Analysis**

Data analysis was done using the IBM SPSS version 26 (SPSS Inc., Chicago, Ill., USA).

## Results

**Fig. 1:**
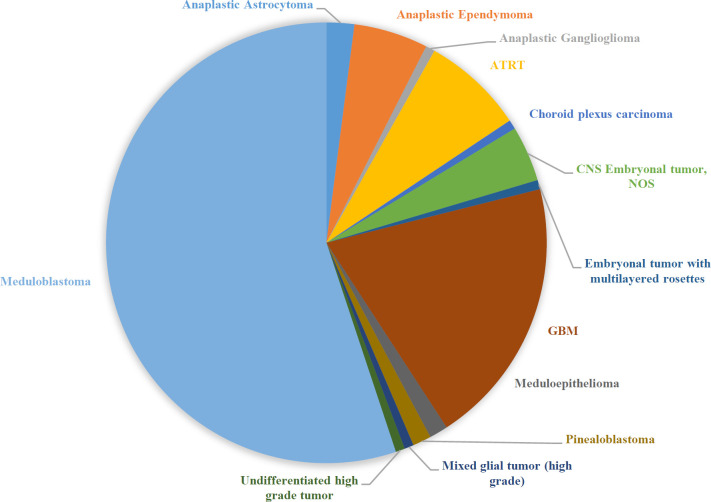
Distribution of different types of high-grade brain tumors in this study

This study was done on Hematoxylin and Eosin (H&E) slides of 147 patients aged 0-17 years with the median age of 8.9 years. These patients were involved with different types of high-grade brain tumors at different areas of the brain. Additionally, the male-to-female ratio was 1.7:1. Disease distribution by the age, gender, and location, as well as the INI1 IHC results has been summarized in Table 1. Besides, the prevalence of different types of high-grade brain tumors has been depicted in Figure 1. Based on the results, medulloblastoma was the most frequent brain tumor in this population.

**Table 1:** Disease distribution in different ages, genders, and locations and the INI1 IHC study results

Disease	Number	Median age	Age range	M/F	INI1	Location
Diffuse astrocytic and oligodendroglial tumors						
Anaplastic astrocytoma	4	13	5-17	1:2	+	Temporal lobe (25%), 3^rd^ ventricle (25%), and frontal lobe (50%)
GBM	29	16	5-17	0.93:1	+	Frontal lobe (59%), temporal lobe (28%), parietal lobe (6.5%), and occipital lobe (6.5%)
Mixed glial tumor (high grade)	1	5	5	1 male	+	Occipital lobe
Ependymal tumors						
Anaplastic ependymoma	8	3.5	6m-15	1:1	+	Temporal lobe (38%), posterior fossa (12.5%), frontal lobe (25.25%), and CP angle (25.25%)
Choroid plexus tumors						
Choroid plexus carcinoma	1	2	2	1 male	+	4^th^ ventricle
Neuronal and mixed neuronal-glial tumors						
Anaplastic ganglioglioma	1	14	14	1 female	+	Posterior fossa
Embryonal tumors						
ATRT	11	2	2m-9	4.5:1	-	Posterior fossa (28%), temporal lobe (28%), CP angle (18%), 4^th^ ventricle (18%), and 3^rd^ ventricle (8%).
Medulloblastoma	81	8	3m-17	2:1	+	Posterior fossa (79%) and 4^th^ ventricle (21%)
Medulloepithelioma	1	2	2	2 males	+	Posterior fossa (50%) and occipital lobe (50
Embryonal tumor with multilayered rossetes	1	1	1	1 male	+	Parietal lobe
CNS embryonal tumor, NOS	6	5.5	3-17	5:1	+	Posterior fossa (33%), temporal lobe (33%), frontal lobe (17%), and occipital lobe (17%)
Undifferentiated high-grade tumor	1	1	1	1 male	+	Temporal lobe
Tumors of pineal region						
Pinealoblastoma	2	6	8m-12	1:1	+	Pineal region
Total	147	8	2m-17	1:7		

Out of the 147 cases, 126 were studied for the INI1 expression using IHC. Unfortunately, the other blocks could not be retrieved. Re-evaluation of the H&E slides caused changes in some diagnoses according to the WHO new classification (12). In addition, more attention was paid to the missed rhabdoid features in some cases. It is worth mentioning that the male-to-female ratio was 1:7, irrespective of the disease type.

Among the 147 cases, 81 had medulloblastoma. In this group, the male-to-female ratio was almost 2. All the cases were placed at the posterior fossa, except for two that were located at the fourth ventricle.

On the H&E slide review, some impressions were changed according to the morphology and INI1 expression and WHO 2021 criteria. All of them except one were confirmed by the INI1 IHC study. Summary of them is seen in Table 2.

**Table 2:** Patients with altered diagnoses according to the morphology and INI1 expression and WHO 2021 criteria.

Number	Initial Diagnosis	Final Diagnosis	INI1	Age	Gender	Tumor Location
1	Medulloblastoma	ATRT	-	1	Male	Posterior Fossa
2	Medulloblastoma	ATRT	-	2	Female	Posterior Fossa
3	Medulloblastoma	ATRT	-	2	Female	Posterior Fossa
4	Anaplastic oligodendroglioma	ATRT	-	3	Male	Posterior Fossa
5	PNET	ATRT	-	1	Male	Posterior Fossa
6	PNET	CNS Embryonal Tumor/NOS	+	2.5	Male	Posterior Fossa
7	PNET	CNS Embryonal Tumor/NOS	Not Performed	3.5	Male	Posterior Fossa

**Fig. 2:**
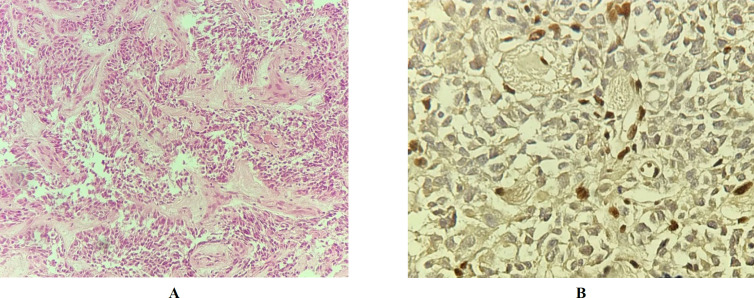
**A) **Patient #1: H&E on medium power shows sheets of the cells with eosinophilic cytoplasm and areas of neuronal differentiation. **B)** Patient #1: INI1 study. Immunostaining with INI1 shows the loss of nuclear expression in the tumor cells with retained expression in intra-tumor blood vessels as an internal positive control.

The first patient was a one-year-old boy who was previously diagnosed with medulloblastoma. In this study, the H&E slides were reviewed and areas with various types of differentiation were detected (like epitheloid and neuronal differentiation). Patchy areas of eosinophilic plasmacytoid cells with moderate cytoplasm and dense chromatin (rhabdoid cells) were found, as well. Therefore, ATRT was suspected. This diagnosis was confirmed by the INI1 IHC. The tumor cells were also immunonegative for INI1. Hence, the diagnosis was changed to ATRT (Figure 2). Unfortunately, the patient died two years after the brain surgery.

**Fig. 3:**
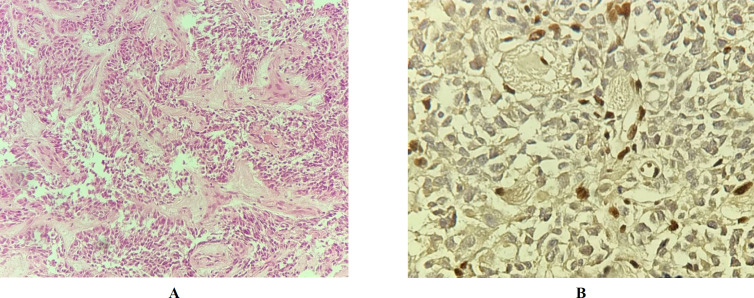
**A)** Patient #2: H& E staining on medium power shows solid sheets of the epitheloid cells with abundant weakly eosinophilic cytoplasm. **B)** Patient #3: H&E staining shows hypocellular zones alternated with the hypercellular areas composed of small blue round cells tufting around the blood vessels.

The second patient was a two-year-old girl with the primary diagnosis of medulloblastoma. Review of the H&E slides revealed solid sheets of the epitheloid cells with abundant weakly eosinophilic cytoplasm and eccentric nuclei with sparse chromatin admixed with the areas of small blue round cells with high mitotic activity and some areas of pseudo-rosette formation. The IHC study for INI1 indicated the loss of nuclear immunoreactivity in the tumor cells and retained staining in the endothelial cells as the positive control. This IHC interpretation confirmed a diagnosis of ATRT for this case (Figure 3A). Unfortunately, the patient died three months after the diagnosis.

The third challenging patient of this study was a two-year old girl with the previous diagnosis of medulloblastoma. The H&E slide review revealed hypocellular areas of the cells with clear cytoplasm and eccentric round nuclei admixed with hyper cellular dense small areas composed of small blue round cells and true and pseudo-rosette formation. No rhabdoid areas were identified. The IHC study for INI1 exhibited the loss of nuclear staining in tumor cells and its retention in the endothelial cells as the positive internal control (Figure 3B). Unfortunately, the patient died two years after the diagnosis.

The other patients diagnosed with medulloblastoma were immunopositive for INI1, as expected (15). 

The classic molecular alterations of astrocytic tumors do not involve INI1, as mentioned in the WHO 2016 and 2021 classification systems (12). Nevertheless, some studies revealed the loss of INI1 expression in glioblastoma, indicating an underlying molecular abnormality was linked to a more aggressive clinical behaviors (16).

The fourth challenging patient was a three-year-old boy who had a glial tumor, with the primary diagnosis of anaplastic oligodendroglioma. The H&E slides review demonstrated solid areas of clear cells with abundant cytoplasm and central nuclei (fried-egg appearance) admixed with the cells with moderate amphophilic cytoplasm and small eccentric nuclei encircling blood vessels. Pseudo-rosette formation was also apparent in some areas. In some fields, there were sheets of cells with pleomorphism, high nuclear cytoplasmic ratio (N/C), and increased mitotic count. This case was immunonegative for INI1 in the present examination. 

**Fig. 4:**
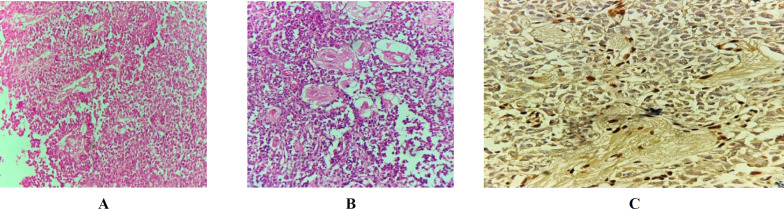
Patient #4: ** A)** H&E staining on low power shows cells with abundant eosinophilic cytoplasm tufting around blood vessels resembling pseudo-rosette formation. **B) **(recurrence): H&E staining shows sheets of cells with eosinophilic cytoplasm and areas of neuronal differentiation at medium power. **C)** (recurrence): Loss of INI1 staining in the tumor cells at medium power.

Due to the tumor relapse, the patient was admitted to the hospital 11 months following the diagnosis. Review of the H&E slides for the second specimen revealed sheets of loosely cohesive cells with eosinophilic cytoplasm and small eccentric nuclei tufting around blood vessels. Some areas of neuronal differentiation and clear cell change were detected, as well. The INI1 IHC study in the second specimen of this case was also negative (Figure 4). This case was reported as ATRT (17) one year after the primary diagnosis when the patient was referred due to the recurrence. Unfortunately, the patient passed away a few months after the second brain surgery.

**Fig. 5:**
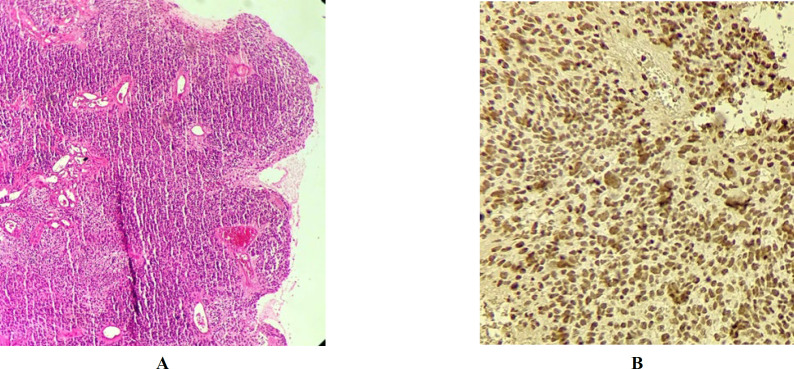
**A)** Anaplastic ependymoma H&E staining shows marked hypercellularity and perivascular rosettes. **B)** INI1 staining of the same tumor shows strong nuclear staining in the tumor cells.

Four cases under the present investigation were diagnosed as anaplastic astrocytoma located in three different areas; i.e., temporal lobe (25%), third ventricle (25%), and frontal lobe (50%). The male-to-female ratio was 1:2. The INI1 IHC study exhibited retained nuclear staining. Furthermore, eight cases were diagnosed with anaplastic ependymoma mostly located at the temporal lobe (38%) followed by the posterior fossa (12.5%), frontal lobe (25.25%), and CP angle (25.25%). The INI1 retention was found in all these cases (Figure 5).

Three cases were previously diagnosed as PNET, but the diagnoses of two of them were changed to embryonal tumor and NOS, according to the WHO new classification system (Table 2).

**Fig. 6:**
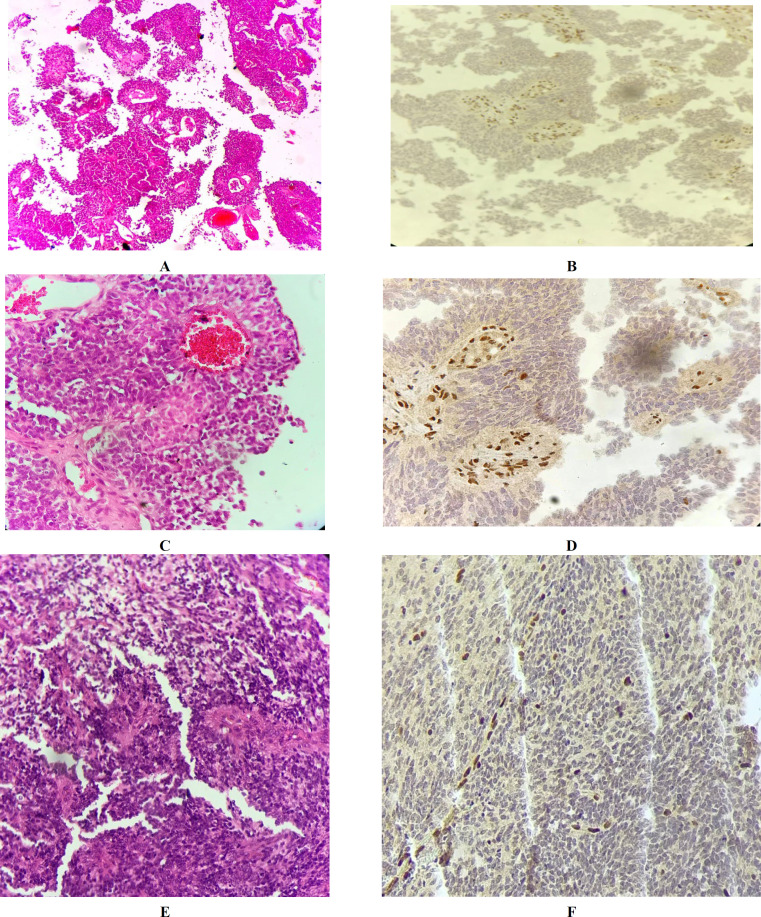
Patient 5:** A)** H&E slides shows sheets and cords of small blue round cells tufting around blood vessels and areas of pseudo-rosette formation (low power). **B)** INI1 IHC study shows the loss of INI1 in the tumor cells (medium power). **C)** High power view: H&E staining shows sheets and cords of small blue cells tufting around blood vessels. **D)** INI1 study at high power **E)** H&E slides (recurrence) shows sheets of small blue round cells tufting around blood vessels. **F)** INI1 IHC study, which is negative in the tumor cells.

The fifth thought-provoking patient was a one-year-old boy previously diagnosed as PNET. The H&E slide review revealed sheets and cords of small blue round cells tufting around blood vessels and areas of pseudo-rosette formation with high mitotic rate and some atypical mitosis. The IHC interpretation for the INI1 revealed the loss of nuclear staining in the tumor cells and the retention of INI1 in the endothelial cells as the positive control. Thus, the diagnosis was changed to ATRT. This patient referred due to the recurrence one year after the primary diagnosis. The second specimen microscopic evaluation revealed alternating areas of hyper- and hypocellularity with heterogeneous appearance. At high power also, there were dense sheets of small blue round cells with a high N/C ratio admixed with the small nests of larger epitheloid cells with amphophilic cytoplasm tufting around blood vessels. The IHC study for INI1 showed the loss of nuclear staining in tumor cells (Figure 6). Unfortunately, the patient passed away five months after the second surgery.

Out of the 147 cases, six had been diagnosed as ATRT. The H&E slides were reviewed and four blocks were retrieved. The INI1 IHC study revealed the loss of INI1 nuclear staining, as expected (10, 18). Finally, 11 ATRT cases were diagnosed according to the slide review and IHC study. 

Two of the cases under the present investigation were diagnosed as pinealoblastoma. These tumors were located at the pineal region. The male-to-female ratio was 1:1. The INI1 IHC study revealed the retention of nuclear staining. One other case was choroid plexus carcinoma (CPC) in a two-year-old boy. Some cases of CPC are immunonegative for INI1 (19), but the IHC study of the present CPC case exhibited retained nuclear staining.

## Discussion

Traditionally, the ATRTs are identified by a triad of clinical manifestations (less than two years old), histological features including presence of the rhabdoid cells (3, 20) defined by eccentric vesicular nuclei, prominent nucleoli, and eosinophilic cytoplasmic inclusions (3, 5, 21), and IHC results showing the expression of vimentin, smooth muscle actin (SMA), epithelial membrane antigen (EMA), and some other markers such as glial fibrillary acidic protein (GFAP), neuronal markers (neurofilament protein and synaptophysin), and cytokeratin (CK) (21, 22). The differential diagnosis of ATRTs includes meduloblastoma, especially large cell/anaplastic variants, poorly differentiated CPC, ependymoma, and other high-grade embryonal tumors, all of which are immunopositive for the INI1 that differentiates them from ATRTs (22, 23). The ATRTs can occur at any place in the CNS. Supratentorial tumors, on the other hand, become more common as people grow older (24).

The INI1 deficiency has been often found to be associated with a more malignant behavior in tumors of different organs (25). Because of the recent discovery of INI1 negative tumors with rhabdoid morphology (9) as well as changes in the WHO classification of the pediatric brain tumors (12), 126 out of the 147 high-grade pediatric brain tumors were evaluated for the INI1 nuclear expression in the current study.

In 2021, Miller et al. hypothesized that high-grade embryonal brain tumor constituted a disease spectrum rather than distinct entities. They found certain PNETS that lacked rhabdoid feature and exhibited the loss of INI1 nuclear staining on IHC (9).

Maysa Al-Hussaini et al. conducted a research on pediatric CNS brain tumors in 2014 and disclosed that rhabdoid features were not primarily detected, at least in some cases. They suggested the performance of the INI1 IHC study on all the embryonal pediatric brain tumors with small cell components in the children aged less than five years to prevent misdiagnosis of the ATRTs (14).

In the present study, the ATRTs involved children between two months and nine years old, with the median age of two years old. A male predominance was also detected. Additionally, five new cases of the ATRT were previously diagnosed as medulloblastoma or PNET. In some of these cases, the diagnosis of ATRT without the IHC study was very challenging. In fact, misdiagnosis seemed to occur due to the lack of experience about divergent patterns in the ATRT. In other words, since this tumor is very rare in comparison with other embryonal brain tumors, diagnostic pitfalls are inevitable. Thus, the INI1 IHC study is highly recommended in embryonal brain tumors in children below two years old so as to distinguish the ATRT from its mimickers.

The main histological features of ATRTs include areas of rhabdoid cells (defined as cells with eccentric vesicular nuclei, prominent nucleoli, and eosinophilic cytoplasmic inclusions), presence of small round cells, and IHC evidence of the divergent differentiation along with mesenchymal, epithelial, glial, or neuronal lineages (22, 26). In the present study, 11 ATRT cases were detected, six of which were primarily diagnosed as ATRT. Some of the cases that were falsely diagnosed as tumors like medulloblastoma had small foci of rhabdoid cells. They might have been misdiagnosed due to pathologists’ lack of experience about the diagnosis of this rare tumor. Furthermore, various types of differentiation including sheets of epitheloid cells and areas with neuronal differentiation were observed in the present cases. Rosette or pseudo-rosette formations were detected, as well. Most of the tumors had at least small foci of rhabdoid cells. In one of the ATRT cases in this study, there were cells with abundant clear cytoplasm, which led to their misdiagnosis as anaplastic oligodendroglioma. Overall, ATRTs have various morphologies and rhabdoid features may not be always prominent. Considering the heterogenecity of the tumor, small streotactic biopsies may not be representative of all tumor parts. The majority of ATRTs are the product of the biallelic inactivation of *SMARCB1* (*INI1*), but they are rarely associated with the inactivation of *SMARCA4* (*BRG1*). Obviously, rare cases with the *SMARCA4* mutation would be INI1 immunopositive, thereby being misleading for pathologists (27, 28). Hence, using both BRG1 and INI1 simultaneously seems to be beneficial for a more definitive diagnosis of ATRT (29) and can be employed in the further investigations.

Some studies have reported the mutations of INI1 in some CPC cases (16). However, considering the low number of CPCs in the current study, this study was not comprehensive about INI1 expression in CPCs. Therefore, a larger number of cases should be examined in the future studies in order to achieve more reliable results.

There are some cases of ATRT that arise on the basis of other CNS tumors including gangliogliomas, pleomorphic xanthoastrocytomas, and high-grade gliomas (30). The tumors evaluated in the present study were mostly primary tumors. In some cases that primary tumor, as well as its recurrence were accessible, they were double checked with respect to the INI1 expression in order to determine the possibility of a secondary mutation in the recurrence of the primary tumor that could lead to a secondary ATRT. They were also checked for the INI1 immunopositive areas. However, all the cases were immunonegative for the INI1 in both primary and recurrent tumors.

## Conclusion

In conclusion, without the INI1 IHC study, the ATRTs may be misdiagnosed with its mimickers due to morphological heterogenecity as well as the lack of experience about this rare but fatal tumor. The ATRT is a high-grade tumor with poor prognosis and needs more intensified chemoradiotherapy in comparison with its mimics like medulloblastoma. The INI1 IHC study can be helpful in the diagnostic pitfalls. Thus, INI1 is recommended to be utilized in the initial IHC panel for the high-grade brain tumors in children, especially those under the age of three years.

## Authors’ Contribution:

Mansoureh Shokripour: designation of the study, review of the slide and analysis. Maryam Mohebbi: collection of data and review of the slides and analysis. Maral Mokhtari: analysis of data, preparing the figures. All the authors contributed in writing the manuscript and approved the final version.

## Ethics Approval and Consent to Participate

This study was approved by the Institutional Review Board of Shiraz University of Medical Sciences, Shiraz, Iran. All the study protocols were in accordance with the guidelines stated in the Declaration of Helsinki. The ethical code of this study was: IR.SUMS.MED.REC.1399.010

## Availability of Data and Material

The present article includes all the information.

## Conflict of Interest

The authors declared no competing interests.
